# Sodium hydrogen sulfide enhances microcirculation and survival in random-pattern skin flaps: An experimental rat study

**DOI:** 10.1016/j.jpra.2026.10.005

**Published:** 2026-10-22

**Authors:** Mustafa Onal, Aysun Ozbay Onal, Mehmet Goktug Esmer, Oguz Kaan Tombul, Merdan Serin

**Affiliations:** a Istanbul Medeniyet University, Department of Plastic, Reconstructive and Aesthetic Surgery, Faculty of Medicine, Turkey; b Istanbul Aydın University, Department of Histology and Embryology, Faculty of Medicine, Turkey; c Experimental Animal Application and Research Center, Maltepe University, Turkey

**Keywords:** Hydrogen sulfide, Sodium hydrogen sulfide, Random-pattern skin flap, Microcirculation, Angiogenesis, Apoptosis, Reconstructive surgery

## Abstract

**Objectives:**

Random-pattern skin flaps remain vulnerable to distal ischemia and partial necrosis despite surgical refinements. Hydrogen sulfide (H₂S) is an endogenous gasotransmitter with cytoprotective and proangiogenic properties. This study evaluated the effects of sodium hydrogen sulfide (NaHS) on flap viability, microvascular perfusion, and histological outcomes in a rat dorsal random-pattern skin flap model.

**Methods:**

Twenty-one male Sprague–Dawley rats were randomized into control, sham, and NaHS groups (n = 7 each). NaHS (200 µg/kg/day) was administered intraperitoneally for 15 days prior to flap elevation. A 9 × 3 cm caudally based dorsal flap was created using the McFarlane model. On postoperative day 7, perfusion was objectively assessed using indocyanine green fluorescence angiography. Inflammation, re-epithelialization, vascularization, and collagen organization were assessed using semi-quantitative scoring, whereas the apoptotic index was determined quantitatively based on TUNEL-positive cell counts.

**Results:**

NaHS significantly increased viable flap area and reduced distal necrosis compared with control and sham groups. Fluorescence imaging demonstrated enhanced distal perfusion and a denser microvascular network in the NaHS group. Histologically, NaHS promoted complete re-epithelialization, organized collagen structure, and markedly increased vascularization (p < 0.01), while significantly decreasing inflammation and apoptosis (p < 0.001).

**Conclusions:**

Sodium hydrogen sulfide improved random-pattern skin flap viability by enhancing microvascular perfusion and attenuating ischemia-induced inflammation and apoptosis. These findings suggest that H₂S donors may represent a potential pharmacologic approach to improve ischemic flap survival in reconstructive surgery.

**Abbreviations**
H₂S: Hydrogen sulfide
NaHS: Sodium hydrogen sulfide
ICG: Indocyanine green
H&E: Hematoxylin and eosin
NO: Nitric oxide
CO: Carbon monoxide
DAB: Diaminobenzidine

## Introduction

Random-pattern skin flaps are among the most commonly used reconstructive techniques in plastic surgery, particularly when axial or perforator-based flaps are unavailable owing to trauma, infection, or previous surgery.^1^ Despite their versatility, distal ischemia and partial necrosis remain major challenges, leading to prolonged wound healing, secondary procedures, and increased morbidity.^2^ Numerous strategies, including pharmacologic agents, ischemic preconditioning, and hyperbaric oxygen, have been explored to improve flap viability; however, an ideal method to consistently enhance distal perfusion has not been established.^3^

Hydrogen sulfide (H₂S), historically regarded as a toxic gas, is now recognized as the third endogenous gasotransmitter, alongside nitric oxide (NO) and carbon monoxide (CO).^4^ It exerts potent cytoprotective, anti-inflammatory, and pro-angiogenic effects by modulating multiple signaling cascades, including the *PI3K/Akt, ERK1/2*, and *VEGF* pathways.^5^ Endogenously, H₂S is synthesized from L-cysteine by cystathionine-β-synthase and cystathionine-γ-lyase, with physiological concentrations contributing to vascular homeostasis and tissue repair.^6^ Experimental studies have demonstrated that exogenous H₂S donors, such as sodium hydrogen sulfide (NaHS), improve microvascular perfusion, limit ischemia-reperfusion injury, and accelerate cutaneous wound healing in diabetic and ischemic models.^7^^,^^8^

The molecular mechanisms underlying these benefits are complex. H₂S induces vasorelaxation by activating ATP-sensitive potassium channels,^9^ enhances angiogenesis through VEGF upregulation,^10^ and attenuates oxidative stress by increasing superoxide dismutase and glutathione peroxidase activity.^11^ Furthermore, it inhibits leukocyte adhesion, reduces pro-inflammatory cytokine release, and suppresses apoptotic signaling, collectively contributing to tissue preservation under hypoxic conditions.^12^ These properties make H₂S a promising therapeutic molecule for improving random-pattern skin flap survival, where microcirculatory compromise and inflammation are the principal causes of necrosis.^13^

Based on these observations, the present experimental study aimed to evaluate the protective effects of NaHS on microcirculation, angiogenesis, and survival of random-pattern skin flaps in rats. We hypothesized that systemic NaHS administration would enhance flap viability by promoting vascular regeneration and mitigating apoptosis and inflammation, thereby improving overall wound healing in a reproducible animal model.

Although hydrogen sulfide donors have been investigated in chronic wound models such as diabetic ulcers, evidence regarding their therapeutic efficacy in random-pattern skin flap models remains scarce. Unlike chronic wounds, flap ischemia involves acute microcirculatory compromise and perfusion gradients, requiring different therapeutic dynamics. Therefore, investigating NaHS in a standardized random-pattern skin flap model may provide novel mechanistic and translational insights into its therapeutic potential in acute ischemic tissue injury relevant to reconstructive surgery.

## Materials and methods

### Ethical statement

All animal procedures were conducted in accordance with the National Institutes of Health Guidelines for the Care and Use of Laboratory Animals and were approved by the Maltepe University Local Ethics Committee for Animal Experiments (approval date: January 30, 2023; protocol no. 2022.12.02). All efforts were made to minimize animal discomfort and reduce the number of animals used.

### Animals and experimental groups

Twenty-one healthy male Sprague–Dawley rats (6 weeks old, weighing 250–300 g, median body weight: 275 g) were obtained from the Maltepe University Laboratory Animal Center. All animals were housed in standard polypropylene cages under controlled environmental conditions (22–24 °C, 12-h light/dark cycle) with ad libitum access to standard chow and water.

The rats were randomly assigned to three experimental groups (n = 7 per group):

1. Control group: Underwent the surgical procedure without receiving any treatment or injection.
2. Sham group: Received intraperitoneal administration of 0.9% saline (1 mL/day) for 15 consecutive days prior to flap elevation.
3. NaHS group: Received intraperitoneal NaHS at a dose of 200 µg/kg/day for 15 consecutive days prior to surgery.

The selected NaHS dose and treatment duration were based on previously published studies demonstrating that this regimen increases endogenous H₂S bioavailability without inducing systemic toxicity.^14^

### Anesthesia and surgical technique

General anesthesia was induced with intraperitoneal ketamine hydrochloride (90 mg/kg) and xylazine hydrochloride (10 mg/kg). After shaving and disinfecting the dorsal surface with povidone-iodine, a 9 × 3 cm random-pattern dorsal skin flap was elevated with its base oriented caudally, following the standardized McFarlane flap model. The flap elevation included the panniculus carnosus and was performed at a uniform depth in all animals. After elevation, the flap was returned to its original bed and secured using 4-0 polypropylene interrupted sutures. All flap dimensions, orientation, and surgical steps were standardized across all groups. Postoperatively, rats were allowed to recover under standard housing conditions, without receiving antibiotics or additional interventions.

All flaps were elevated to include the panniculus carnosus at a consistent thickness to ensure reproducibility. The procedure was performed by a single experienced surgeon, and flap elevation was standardized at a consistent thickness to ensure reproducibility. The surgeon was not blinded to group allocation during surgery but was not involved in outcome assessment. The flap design was based on the standardized dorsal random-pattern flap model originally described by McFarlane.^15^

### Fluorescence microangiography

On postoperative day 7, all animals underwent fluorescence microangiography to evaluate flap microcirculation. Under general anesthesia, the flap was re-elevated along the previous incision lines. Indocyanine green (ICG) (10 mg dissolved in 10 mL saline) was freshly prepared, and 0.1 mL was administered via intracardiac injection. ICG fluorescence imaging was initiated immediately after administration, upon visualization of the fluorescence signal within the flap vasculature, using the SPY Elite® fluorescence imaging system (Stryker, USA). The same anatomical region of the flap was evaluated and documented in all animals to ensure consistency across the experimental groups.

Fluorescence signals were recorded from the proximal to distal regions of the flap. Perfusion quality was assessed qualitatively by three blinded plastic surgeons. The presence of continuous fluorescence extending to the distal flap margin was interpreted as evidence of improved tissue perfusion.

Perfusion quality was semi-quantitatively graded by three blinded plastic surgeons using a 4-point scale:

0 = no perfusion,

1 = minimal proximal perfusion,

2 = partial distal perfusion,

3 = complete perfusion extending to distal flap margins.

The mean score of the three blinded observers was used for statistical analysis.

### Tissue harvesting and histopathology

After imaging, all animals were euthanized with an overdose of ketamine followed by cervical dislocation to ensure complete cessation of vital signs. For histopathological evaluation, tissue specimens were consistently obtained from the distal portion of each flap at the macroscopically identified transition between necrotic and viable tissue. The same sampling approach was applied to all animals and experimental groups. The specimens were fixed in 10% neutral-buffered formalin for 48 h.

After fixation, tissues were embedded in paraffin, sectioned at a thickness of 5 µm, and stained with H&E and Masson’s trichrome. Histopathological examination was performed using light microscopy.

### Histopathological evaluation

Histological parameters were evaluated using a semi-quantitative scoring system. Four criteria were assessed: inflammation, re-epithelialization, vascularization, and collagen deposition. Each parameter was graded on a 0–3 scale (0 = absent, 1 = mild, 2 = moderate, 3 = marked).

Re-epithelialization and vascularization were assessed as the percentage of tissue exhibiting newly formed epithelium or neovascularization, respectively. Collagen organization was assessed using Masson’s trichrome staining, and inflammatory cell infiltration was quantified by counting inflammatory cells per high-power field (× 400).^16^

### TUNEL assay

Apoptotic activity was evaluated using a TUNEL (terminal deoxynucleotidyl transferase dUTP nick-end labeling) assay with a commercial kit (My BioSource, USA; Cat. No. MBS2557027). After deparaffinization and rehydration, sections were permeabilized with Proteinase K for 30 min and treated with 3% hydrogen peroxide to block endogenous peroxidase activity. The slides were then incubated with the TdT reaction mixture for 1 h at 37 °C, developed with diaminobenzidine (DAB), and counterstained with Gill’s hematoxylin.

Apoptotic activity was quantified in five randomly selected high-power fields per section. The apoptotic index was calculated as the percentage of TUNEL-positive nuclei relative to the total number of nuclei.

### Statistical analysis

Statistical analyses were performed using GraphPad Prism version 10.0.2 (GraphPad Software, San Diego, CA, USA). Continuous variables were presented as mean ± standard deviation (SD). Normality of data distributions was assessed using the Shapiro–Wilk test. Normally distributed variables were analyzed using one-way analysis of variance (ANOVA) followed by Tukey’s multiple comparison test, whereas non-parametric data were evaluated using the Kruskal–Wallis test with Dunn’s post-hoc analysis. A p-value < 0.05 was considered statistically significant.

## Results

### Macroscopic findings

Macroscopic evaluation demonstrated differences in flap viability among the groups. In the Control group, necrosis developed in the distal portion of the flaps early after surgery and persisted throughout the 6-day observation period without any reduction in size. The necrotic areas showed a dark, non-perfused appearance, whereas the proximal regions remained pink and viable.

The Sham group exhibited findings similar to the Control group. Despite receiving intraperitoneal saline for 15 days before flap elevation, no improvement in distal flap survival was observed. The necrotic zones remained stable in both size and appearance until postoperative day 6.

In contrast, the NaHS-treated group showed markedly smaller necrotic areas with improved distal perfusion. By postoperative day 6, the viable tissue extended further toward the distal end, and the overall extent of necrosis was substantially reduced compared with both Control and Sham groups. These observations indicate that preoperative NaHS administration (200 µg/kg/day for 15 days) provided a clear protective effect on flap viability (Fig. 1 and Supplementary Fig. S1).

**Fig. 1:**
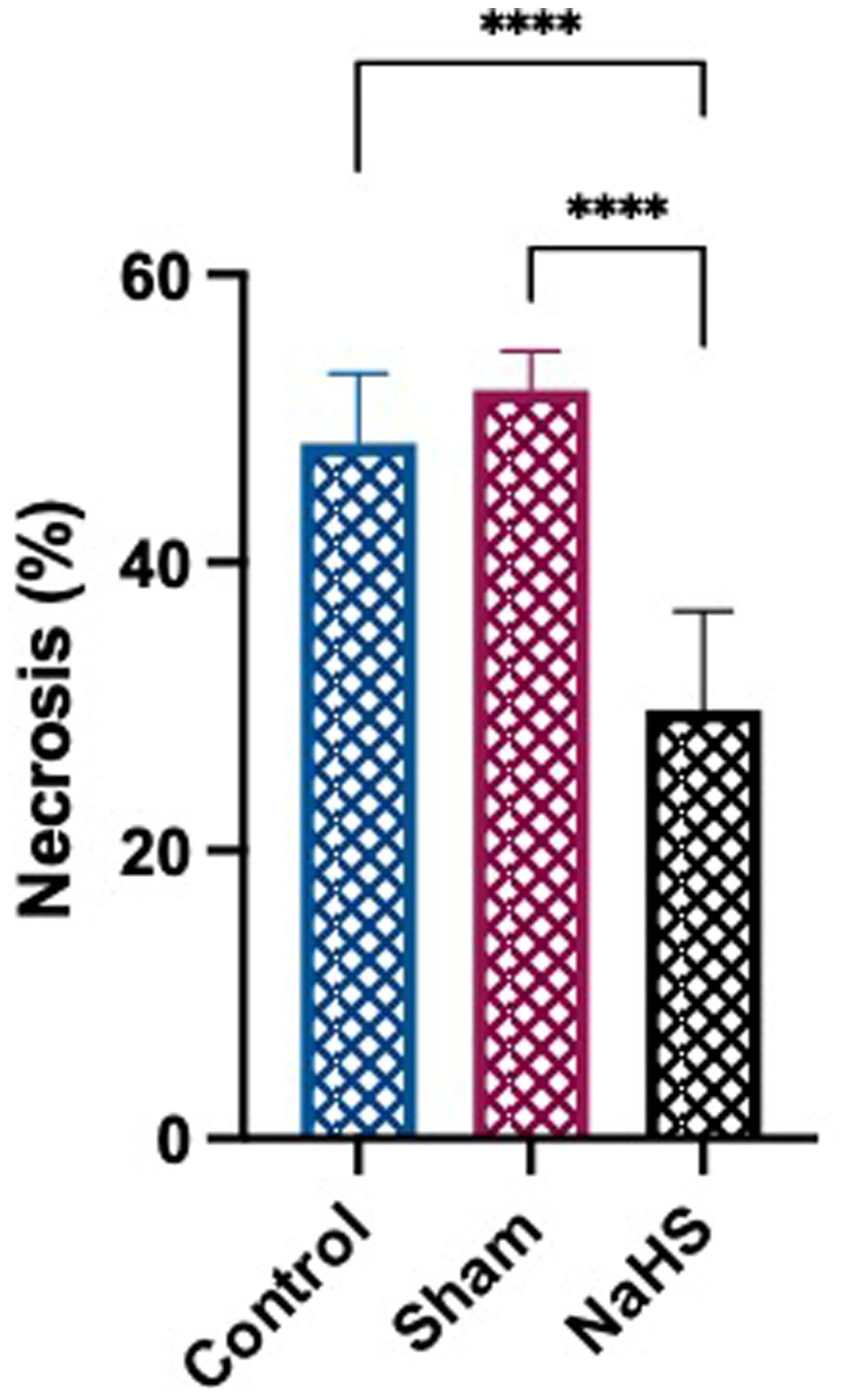
Quantitative necrosis ratios of dorsal skin flaps in all groups. ****p < 0.0001 NaHS = sodium hydrogen sulfide; Sham = physiological saline.

### Microangiographic findings

Fluorescent microangiography on postoperative day 7 revealed marked differences in flap perfusion among the groups. In the Control group, the microvascular network appeared sparse, with limited branching and perfusion confined to the proximal segments of the flap. The Sham group demonstrated similarly reduced perfusion and poor distal filling, comparable to the Control animals.

In contrast, the NaHS-treated group displayed a markedly more extensive and continuous fluorescence pattern, with fluorescent signals extending toward the distal margins of the flap, indicating improved tissue perfusion. Blinded evaluation by three independent plastic surgeons consistently confirmed superior distal perfusion in the NaHS group compared with both Control and Sham groups (Fig. 2).

**Fig. 2:**
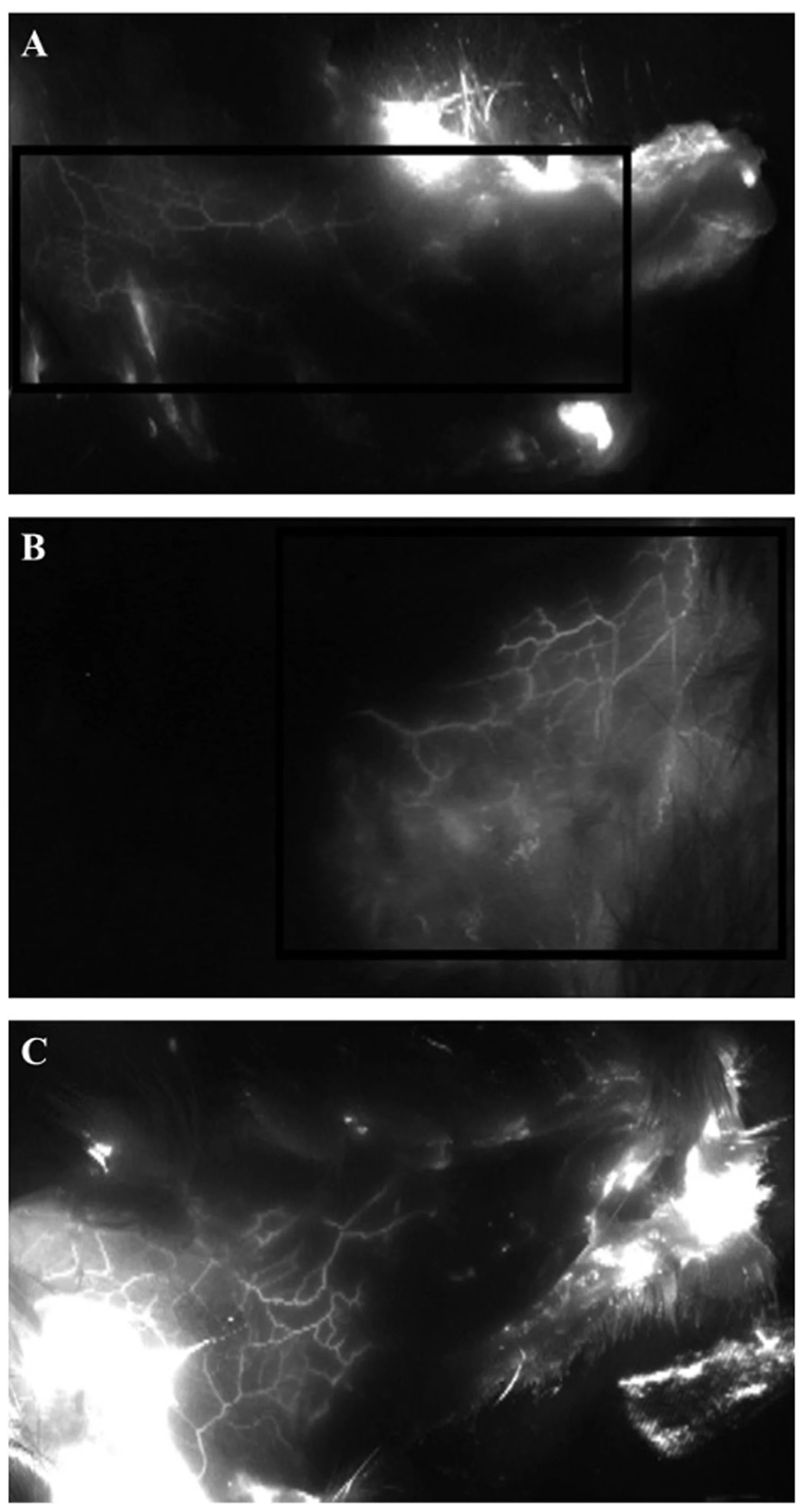
Representative fluorescence microangiographic images of dorsal random-pattern skin flaps in the experimental groups on postoperative day 7. **(A)** Control group showing limited tissue perfusion, with fluorescence signals confined predominantly to the proximal region of the flap. **(B)** Sham group showing a fluorescence perfusion pattern comparable to that of the Control group, with limited distal perfusion. **(C)** NaHS-treated group showing a more extensive fluorescence perfusion pattern, with fluorescence signals extending toward the distal flap region, indicating improved tissue perfusion compared with the Control and Sham groups. NaHS; sodium hydrogen sulfide.

Quantitative perfusion scores were significantly higher in the NaHS group compared with Control and Sham groups (p < 0.01).

### Histopathological findings

Microscopic evaluation of hematoxylin–eosin and Masson’s trichrome–stained sections demonstrated clear histological differences among the groups. In the Control and Sham groups, the epidermis was absent, and immature granulation tissue predominated. Dense infiltration of polymorphonuclear leukocytes and lymphocytes, interstitial fibrin deposition, vascular congestion, and edema were evident. Dermal immaturity was characterized by adipocyte accumulation and disrupted connective tissue organization.

In contrast, the NaHS-treated group exhibited complete re-epithelialization with restoration of epidermal continuity, along with an organized dermal architecture containing well-formed vascular structures and adnexal elements, including sebaceous glands and hair follicles. Inflammatory infiltration was markedly reduced, and collagen bundles appeared more regularly aligned.

Quantitative histopathological scoring supported these findings. Mean inflammation scores were significantly higher in both the Control and Sham groups than in the NaHS group (p < 0.0001). Re-epithelialization scores were significantly greater in NaHS-treated rats compared with Control and Sham animals (p < 0.0001). Vascularization scores followed a similar pattern, being significantly elevated in the NaHS group relative to both Control (p = 0.0012) and Sham (p = 0.0044). Moreover, collagen deposition assessed by Masson’s trichrome staining was markedly increased in the NaHS group compared with the Control and Sham groups (p = 0.0014).

Overall, these histopathological findings indicate that NaHS administration enhanced structural integrity, vascular proliferation, and collagen organization while substantially reducing inflammation. Representative H&E- and Masson's trichrome-stained sections are presented in Fig. 3, Fig. 4, respectively, whereas the corresponding quantitative histopathological and collagen organization scores are provided in Supplementary Figs. S2 and S3.

**Fig. 3:**
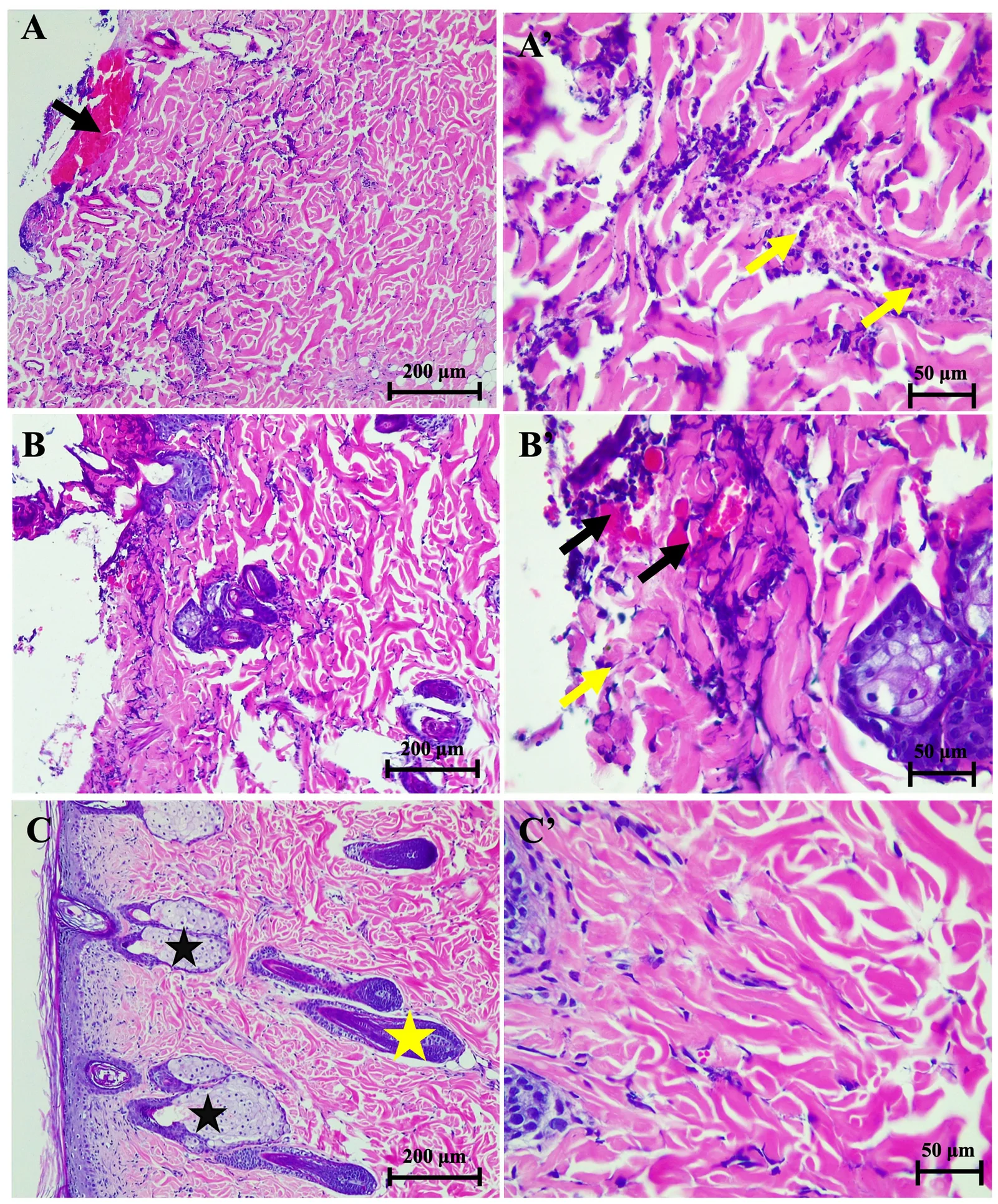
Histopathological evaluation of dorsal random-pattern skin flaps. Representative hematoxylin–eosin (H&E)-stained sections of the experimental groups. (A–A′) Control group showing marked vascular congestion (black arrows), dense inflammatory cell infiltration (yellow arrows), and adipocyte accumulation within immature granulation tissue. (B–B′) Sham group showing prominent inflammatory cell infiltration, vascular congestion, and disrupted dermal architecture. (C–C′) NaHS-treated group showing complete re-epithelialization, organized dermal architecture, preserved sebaceous glands (black star), hair follicles (yellow star), and capillary structures.

**Fig. 4:**
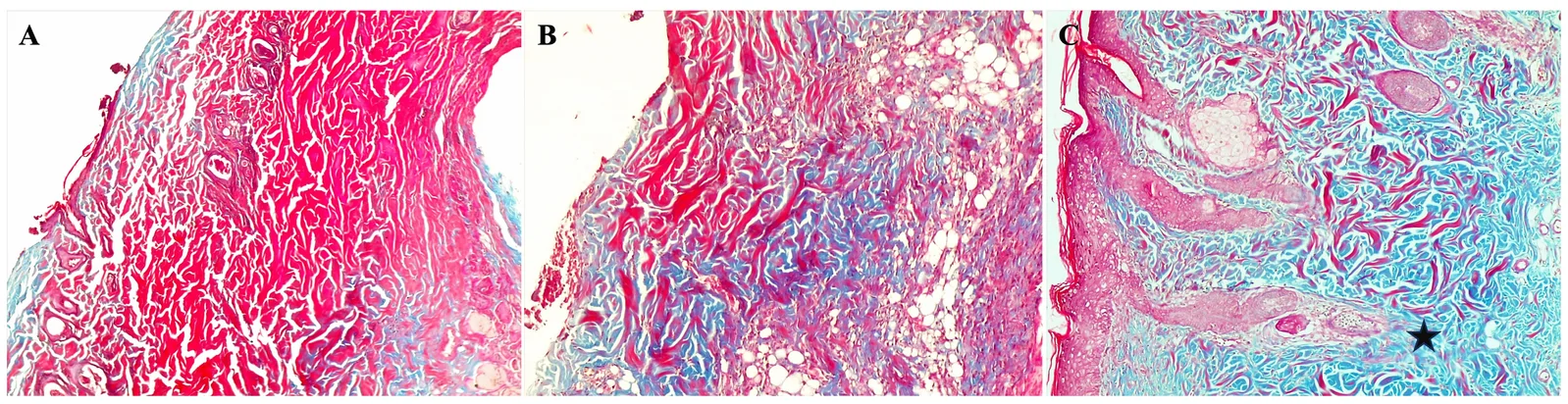
Representative Masson's trichrome-stained sections of the experimental groups. (A) Control group showing disorganized and sparsely distributed dermal collagen fibers with limited structural integrity. (B) Sham group showing irregular collagen organization comparable to the Control group, with areas of adipocyte accumulation. (C) NaHS-treated group showing well-organized collagen bundles (blue staining, black star) with preserved epidermal architecture and adnexal structures, including hair follicles and sebaceous glands.

### TUNEL findings

Apoptotic activity within the flap tissues was assessed using the TUNEL assay. Numerous TUNEL-positive nuclei were observed in fibroblasts, adipocytes, endothelial cells, and inflammatory cells in both the Control and Sham groups, indicating extensive apoptotic injury. In contrast, the NaHS-treated group demonstrated markedly fewer TUNEL-positive cells, accompanied by preserved epidermal and dermal architecture.

Quantitative analysis showed that the apoptotic index was significantly lower in the NaHS group compared with both the Control and Sham groups (p < 0.0001). No significant difference was detected between the Control and Sham animals.

Assessment of necrosis ratios revealed a similar pattern. The mean necrotic area percentage was significantly reduced in the NaHS-treated flaps relative to the Control and Sham groups (p < 0.0001). Overall, these findings confirm that NaHS markedly attenuated apoptosis and necrosis while preserving tissue viability and histological integrity. Representative TUNEL-stained sections are presented in Fig. 5, whereas quantitative apoptotic index data are provided in Supplementary Fig. S4.

**Fig. 5:**
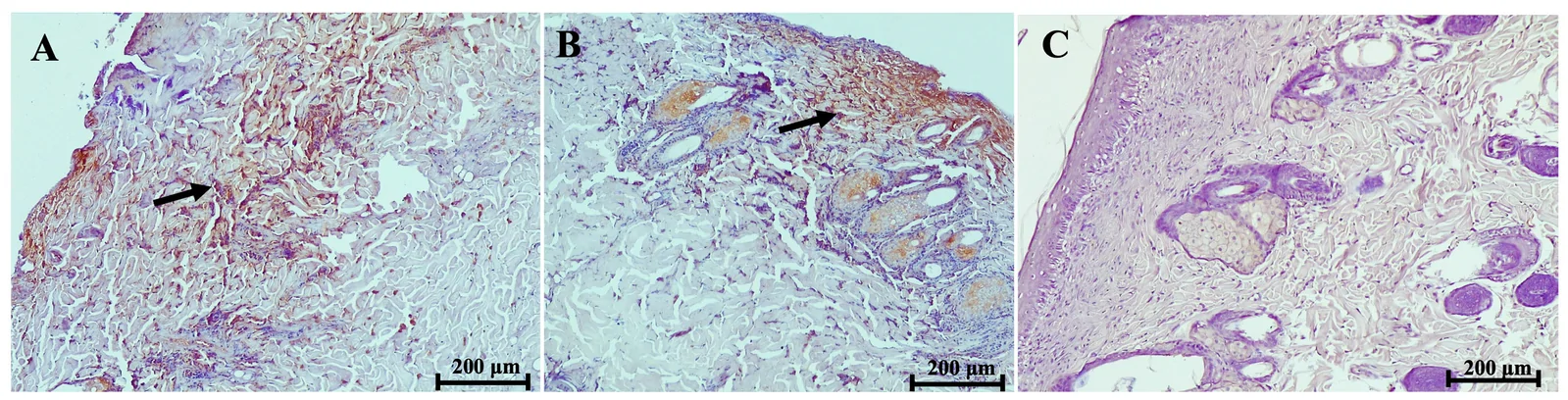
Representative TUNEL-stained sections of the experimental groups. (A–A′) Control group showing numerous TUNEL-positive apoptotic cells (black arrows) within the dermal connective tissue and surrounding vascular structures. (B–B′) Sham group showing persistent apoptotic cells localized predominantly within fibroblasts and endothelial cells of the dermis. (C–C′) NaHS-treated group showing fewer TUNEL-positive cells with preserved epidermal and dermal architecture.

## Discussion

In this experimental rat model of dorsal random-pattern skin flaps, NaHS markedly improved overall flap survival. NaHS-treated flaps demonstrated significantly reduced distal necrosis and enhanced tissue viability, consistent with an improved microcirculatory environment. Correspondingly, fluorescence microangiography confirmed superior distal perfusion and a more extensive fluorescence perfusion pattern in NaHS-treated animals, indicating improved tissue perfusion.

Cellular and structural findings further corroborated these observations. The NaHS group exhibited substantially fewer apoptotic cells and less necrotic tissue, along with better preserved epidermal and dermal architecture. Histological evaluation revealed more organized collagen deposition and reduced inflammatory cell infiltration, suggesting attenuation of ischemia-induced tissue damage.

Overall, these findings indicate that exogenous H₂S confers significant cytoprotective effects in compromised skin flaps. NaHS appears to promote flap survival through a combination of mechanisms, including improved tissue perfusion, enhanced histological vascularization, suppression of apoptosis, and preservation of extracellular matrix integrity. These findings support the potential of NaHS as a pharmacologic adjunct for enhancing the viability of random-pattern skin flaps.

The protective effects observed with NaHS are consistent with well-established H₂S-mediated cytoprotective mechanisms described in the literature.^4^^,^^17^ As the third endogenous gasotransmitter alongside nitric oxide (NO) and carbon monoxide (CO), H₂S regulates multiple signaling pathways that promote cell survival under ischemic stress. H₂S induces vasodilation and enhances microcirculatory perfusion, consistent with the improved distal blood flow observed in the NaHS-treated flaps in the present study. Previous studies have also demonstrated that H₂S activates pro-angiogenic signaling pathways, including VEGF-related mechanisms, providing a plausible biological explanation for the enhanced histological vascularization observed in NaHS-treated tissues.^7^^,^^17^

In addition, H₂S has been widely reported to exert antioxidant and anti-inflammatory effects.^18^ These properties are consistent with the reduced inflammatory cell infiltration and improved dermal organization observed histologically in our NaHS-treated animals. Furthermore, H₂S has been shown to modulate apoptotic signaling by promoting anti-apoptotic pathways and suppressing pro-apoptotic responses,^19^ which is consistent with the significantly lower apoptotic indices identified in our study. Overall, these mechanisms provide a biologically plausible explanation for the cytoprotective effects observed in NaHS-treated flaps.

These mechanistic insights and histological observations suggest that H₂S enhances flap survival through multiple complementary processes, including improved tissue perfusion, enhanced histological vascularization, and attenuation of ischemia-induced cellular injury, thereby creating a more favorable microenvironment for tissue viability in random-pattern flaps.^18^ These findings further support the concept that modulation of endogenous gasotransmitter pathways may serve as a promising therapeutic strategy for optimizing outcomes in ischemic skin flaps.

Our findings are consistent with a growing body of preclinical evidence demonstrating that gasotransmitters and various pharmacologic agents can enhance flap survival. In a comparable random-pattern flap model, apigenin improved flap viability and microvascular perfusion while mitigating oxidative and inflammatory injury—findings that closely parallel observed with NaHS in the present study.^20^ Similarly, cordycepin has been shown to enhance angiogenesis, reduce oxidative stress, and limit apoptosis through autophagy-related mechanisms in ischemic flaps.^21^

Earlier pharmacological studies have also investigated dimethyl sulfoxide (DMSO) as a strategy to improve skin flap viability. Topical DMSO combined with hydrogen peroxide was shown to significantly increase transcutaneous oxygen tension and survival in random skin flaps, with free-radical scavenging proposed as a potential contributor to this protective effect in addition to vascular mechanisms.^22^ Although DMSO and NaHS are chemically distinct sulfur-containing compounds and their mechanisms of action cannot be directly equated, these observations suggest that their protective effects may converge at the functional level through modulation of oxidative injury and microvascular function. In this context, the improved perfusion and flap survival observed with NaHS in the present study may share certain functional features with those previously reported for DMSO-based treatment, without implying a common molecular mechanism. Further studies are needed to clarify the potential mechanistic relationship between these sulfur-containing interventions and their respective contributions to flap protection.

Gas-based therapeutic approaches, including hydrogen sulfide donors, have demonstrated beneficial effects across multiple experimental flap models. For instance, a slow-releasing H₂S donor significantly decreased necrosis and improved microcirculation in an axial-pattern ear flap model in mice. Similarly, continuous hydrogen (H₂) delivery improved flap survival and attenuated cellular injury in a rat ischemia–reperfusion mode.^23^ These findings are consistent with the reduced apoptosis and preserved tissue architecture observed in the NaHS-treated group in the present study.

Comparable protective effects have also been reported with other gasotransmitters. A dual CO/NO-releasing hydrogel has been shown to maintain perfusion and support distal revascularization in rat skin flaps while reducing inflammatory tissue damage.^24^ These findings suggest that interventions that enhance endogenous NO, CO, or H₂S signaling tend to converge on similar biological outcomes, including improved blood flow, enhanced vascularization, and attenuation of ischemia–reperfusion injury.

The present study demonstrates that NaHS improves perfusion, reduces cellular injury, and supports tissue organization in a random-pattern flap model. These findings are consistent with the existing evidence supporting the role of H₂S in flap preservation and further emphasize the importance of improving vascularization while limiting ischemic tissue damage to optimize flap survival.

The present findings have important implications for reconstructive surgery. Flap necrosis remains a persistent challenge, often resulting in reoperations, prolonged wound care, and increased morbidity.^25^ Demonstrating that an H₂S-releasing agent enhanced flap tolerance to ischemia suggests that pharmacologic supplementation could represent a practical adjunct for improving flap viability in clinical settings. This concept aligns with the emerging paradigm of pharmacologic flap optimization, in which agents targeting vasodilation, angiogenesis, oxidative stress, and apoptotic pathways have shown encouraging results.^25^^,^^26^ H₂S donors are particularly compelling because they simultaneously modulate several of these mechanisms, functioning as vasodilators, pro-angiogenic mediators, and cytoprotective molecules.

From a clinical perspective, these findings support the potential development of perioperative ischemic-flap salvage strategies in which an H₂S donor—such as NaHS or a slow-release analog—could be administered systemically or locally to compromised flaps. By improving tissue perfusion and reducing ischemia–reperfusion injury during the early postoperative period, H₂S-based therapies may increase flap viability and reduce the risk of distal flap necrosis. This approach parallels existing interventions such as hyperbaric oxygen therapy or nitroglycerin paste, yet H₂S may offer a more targeted molecular strategy for mitigating ischemic damage. Future studies should investigate optimized delivery systems, such as injectable hydrogels or H₂S-releasing wound dressings, to maximize local therapeutic efficacy while minimizing systemic exposure.^25^^,^^27^^,^^28^

This study has several limitations. First, the relatively small sample size and the use of a rodent random-pattern flap model limit the generalizability of the findings. Rat skin contains a well-developed panniculus carnosus and a denser subdermal vascular plexus than human skin, whereas human flaps rely more heavily on perforator-based circulation. Moreover, the experimental flaps were repositioned in their original anatomical location without the tension, rotation, or shear forces frequently encountered in clinical practice. Second, the 7-day follow-up period represents an early assessment of flap survival and does not capture long-term remodeling, vascular maturation, or delayed necrosis. Third, molecular analyses of key pro-angiogenic and oxidative stress-related pathways, including VEGF, HIF-1α, and endogenous antioxidant enzymes, were not performed and could have provided deeper mechanistic insight into the observed effects of NaHS. Finally, only preoperative systemic NaHS administration was evaluated, and postoperative dosing or localized delivery strategies were not investigated.

From a translational perspective, several steps are required before H₂S-based therapy can be considered for clinical flap preservation. First, clinically applicable local delivery strategies, including topical formulations, injectable hydrogels, or controlled-release H₂S wound dressings, should be evaluated to optimize local efficacy while minimizing systemic exposure. Second, dose–response relationships, treatment timing, pharmacokinetics, and safety should be systematically established. Third, efficacy should be validated in larger animal models that more closely reproduce human skin vascular anatomy and clinically relevant flap conditions. Finally, if efficacy and safety are confirmed in these models, well-designed clinical studies will be required to determine the feasibility and therapeutic value of H₂S-based interventions in reconstructive surgery.

## Conclusion

In this experimental random-pattern skin flap model, systemic NaHS administration improved flap survival and microcirculatory perfusion while reducing ischemia-associated apoptosis and inflammation and preserving tissue architecture. These findings support the potential of H₂S-based strategies for flap preservation. Further studies using clinically applicable delivery systems, optimized dosing regimens, and larger translational models are required before clinical application can be considered.

## Declarations

### Ethical approval

All experimental procedures were approved by the Maltepe University Local Ethics Committee for Animal Experiments (Approval date: January 30, 2023; Protocol No: 2022.12.02).

### Clinical trial number

Not applicable

### Funding

This research received no specific grant from any funding agency in the public, commercial, or not-for-profit sectors.

### Authors’ contributions

Mustafa Onal: Study conception and design; surgical procedures; data collection; interpretation of results; manuscript drafting and critical revision.

Aysun Ozbay Onal: Histological and immunohistochemical analyses; data interpretation; manuscript writing, editing, and final approval.

Mehmet Goktug Esmer: Assistance with experimental procedures; support in data collection and laboratory workflow.

Oguz Kaan Tombul: Animal model preparation, animal care, experimental assistance; contribution to data collection and methodological support.

Merdan Serin: Statistical analysis; data interpretation; revision of the manuscript for important intellectual content.

All authors read and approved the final manuscript.

Supplementary Figure Legends

**Supplementary Fig. S1.** Representative macroscopic appearance of dorsal random-pattern skin flaps in the experimental groups on postoperative days 1, 3, and 7. Necrotic regions are outlined with red dashed lines to facilitate visual assessment of the extent of flap necrosis.

Control group (A–C): Progressive distal flap necrosis with persistent ischemic changes throughout the observation period.

Sham group (D–F): Macroscopic findings comparable to the Control group, with no apparent improvement in distal flap viability.

NaHS group (G–I): Reduced distal necrosis and improved flap viability compared with the Control and Sham groups.

**Supplementary Fig. S2.** Quantitative histopathological scores of the experimental groups for (A) inflammation, (B) re-epithelialization, and (C) vascularization. Compared with the Control and Sham groups, the NaHS group showed reduced inflammation and increased re-epithelialization and vascularization. Data are presented as mean ± SD. Asterisks indicate statistically significant differences (**p < 0.01).

**Supplementary Fig. S3.** Quantitative collagen organization scores of the experimental groups. Data are presented as mean ± SD. Asterisks indicate statistically significant differences (**p < 0.01).

**Supplementary Fig. S4.** Quantitative apoptotic index of the experimental groups. Data are presented as mean ± SD. Asterisks indicate statistically significant differences (****p < 0.0001).

## Declaration of competing interest

The authors declare that they have no competing interests.

## Data Availability

All data supporting the findings of this study are included in this article. Additional raw data are available from the corresponding author upon reasonable request.
