# Supplementary material for: Sodium hydrogen sulfide enhances microcirculation and survival in random-pattern skin flaps: An experimental rat study
Source: JPRAS Open. 2026 Oct 22;53:27–36. doi: 10.1016/j.jpra.2026.10.005 (PMC13721927; doi:10.1016/j.jpra.2026.10.005)
Supplement: Supplementary file 1 [file mmc1.docx]

**ARRIVE Essential 10 Checklist**

**Manuscript title:** Sodium Hydrogen Sulfide Enhances Microcirculation and Survival in Random Skin Flaps: An Experimental Rat Study

**Study design**

**1a.** Three experimental groups were compared: Control, Sham, and NaHS groups (n = 7 per group).

**1b.** The individual animal was the experimental unit.

**Sample size**

**2a.** A total of 21 animals were used. Seven rats were allocated to each experimental group: Control, Sham, and NaHS groups (n = 7 per group).

**2b.** A formal power analysis could not be performed due to insufficient and inconsistent variance data available in the literature for this experimental model. Therefore, the sample size was determined using the Resource Equation Method in accordance with the 3R principle (Reduction). The E value was calculated as follows: E = total number of animals − total number of groups = 21 − 3 = 18. As this value falls within the recommended range of 10–20, seven animals per group were considered appropriate.

**Inclusion and exclusion criteria**

**3a.** Healthy male Sprague–Dawley rats aged 6 weeks and weighing 250–300 g were eligible for inclusion. Animals showing signs of illness, injury, or abnormal general health status were excluded. These criteria were established a priori.

**3b.** No animals, experimental units, or data points were excluded from the analysis.

**3c.** All analyses included seven animals per group (n = 7).

**Randomisation**

**4a.** Animals were allocated to the Control, Sham, and NaHS groups using simple randomization.

**4b.** To minimize potential confounders, all flap dimensions, orientations, and surgical procedures were standardized across groups. All surgical procedures were performed by a single experienced surgeon to minimize inter-operator variability.

**Blinding**

**5.** The surgical procedures were performed with knowledge of group allocation. Outcome assessment was conducted by three plastic surgeons blinded to group allocation.

**Outcome measures**

**6a.** The outcome measures included flap survival and necrosis, microvascular perfusion, histopathological parameters (inflammation, re-epithelialization, vascularization, and collagen deposition), and apoptotic index.

**6b.** No single primary outcome measure was used to determine the sample size, as the sample size was determined using the Resource Equation Method.

**Statistical methods**

**7a.** Statistical analyses were performed using GraphPad Prism version 10.0.2. Normally distributed data were analyzed using one-way analysis of variance (ANOVA) followed by Tukey’s multiple comparison test, whereas non-parametric data were analyzed using the Kruskal–Wallis test followed by Dunn’s post-hoc test. A p-value < 0.05 was considered statistically significant.

7b. Normality of data distributions was assessed using the Shapiro–Wilk test. When the assumption of normality was met, parametric tests were used; when it was not met, non-parametric tests were applied.

**Experimental animals**

**8a.** Twenty-one healthy male Sprague–Dawley rats, aged 6 weeks and weighing 250–300 g (median body weight: 275 g), were used.

**8b.** The animals were obtained from the Maltepe University Laboratory Animal Center. All animals were healthy and had no previous experimental procedures. The animals were not genetically modified.

**Experimental procedures**

**9a.** The Control group underwent flap surgery without treatment or injection. The Sham group received intraperitoneal 0.9% saline (1 mL/day), and the NaHS group received intraperitoneal NaHS (200 µg/kg/day). Under general anesthesia, a standardized 9 × 3 cm caudally based dorsal random-pattern skin flap was elevated and sutured back to its original bed.

**9b.** Saline or NaHS was administered once daily for 15 consecutive days before flap elevation. Fluorescence microangiography was performed on postoperative day 7, followed by tissue harvesting for histopathological and TUNEL analyses.

**9c.** The animals were obtained from and housed at the Maltepe University Laboratory Animal Center under controlled environmental conditions (22–24 °C, 12-hour light/dark cycle), with ad libitum access to standard chow and water. A specific acclimatisation period was not reported.

**9d.** The NaHS dose and treatment duration were selected based on previous studies demonstrating increased H₂S bioavailability without systemic toxicity. The surgical procedure was based on the standardized McFarlane random-pattern flap model to provide a reproducible model of flap ischemia and necrosis.

**Results**

**10a.** Summary/descriptive statistics for each experimental group, including measures of variability where applicable, are presented in the Results section and corresponding figures.

**10b.** Not applicable; effect sizes and confidence intervals were not calculated.
